# The Impact of HPV Female Immunization in Italy: Model Based Predictions

**DOI:** 10.1371/journal.pone.0091698

**Published:** 2014-03-11

**Authors:** Giorgio Guzzetta, Luca Faustini, Donatella Panatto, Roberto Gasparini, Piero Manfredi

**Affiliations:** 1 Trento Rise, Trento, Italy; 2 Fondazione Bruno Kessler, Trento, Italy; 3 Istat Toscana, Florence, Italy; 4 Dipartimento di Scienze della Salute, Università di Genova, Genoa, Italy; 5 Dipartimento di Economia e Management, Università di Pisa, Pisa, Italy; Georgetown University, United States of America

## Abstract

The Human Papillomavirus (HPV) is a sexually transmitted virus that causes cervical cancer. Since 2008 a vaccination program targeting 12-year-old girls has been initiated in Italy, backing up the cervical screening program already active since 1996. We propose a mathematical model of HPV transmission dynamics with the aim of evaluating the impact of these prevention strategies. The model considers heterosexual transmission of HPV types 16 and 18, structured by sex, age and sexual activity level, where transition to sexual activity is explicitly modeled from recent survey data. The epidemiological structure is a hybrid SIS/SIR, where a fraction of individuals recovering from infection develops permanent immunity against reinfection. Infections may progress to cervical lesions and cancer and heal spontaneously or upon treatment. Women undergoing hysterectomy (either after treatment of HPV lesions or by other causes) also transmit HPV infection. The model fits well both the age-specific prevalence of HPV infections and the incidence of cervical cancers in Italy, and accurately reproduces the decreasing trend in cancer incidence due to the introduction of the screening program. The model predicts that if the screening coverage is maintained at current levels, even in the absence of vaccination, such trend will continue in the next few decades, eventually plateauing at 25% below the current level. The additional initiation of routine vaccination targeting 12-year-old girls will further reduce cervical cancer incidence by two thirds at equilibrium, under realistic assumptions of 70% coverage and a duration of protective immunity of 50 years. If catch-up immunization of 25-year-old women at first cervical screening is also introduced, about 3,000 cervical cancer cases overall can be averted, corresponding to 9.6% of all cases expected in the scenario without catch-up. We conclude that HPV vaccination in addition to cervical screening will significantly reduce the burden of cervical cancer in Italy.

## Introduction

Many types of human papillomavirus (HPV) are known. Of these, about 50 have a high tropism for the ano-genital mucosa and are sexually transmitted. According to the most recent classification of the International Agency for Research on Cancer (IARC) 12 genotypes are defined as high-risk (HR) oncogenic, 13 are classified as “probable” and “possible” oncogenic risk and the others are classified as “non-carcinogenic” [1]. The HR HPV genotypes are responsible for the large majority of cervical cancers [2] and for a significant proportion of cancers in other body districts [3], [4]. Upon infection, HR HPVs replicate within squamous epithelial cervical cells and in other epithelial cells of the ano-genital mucosa, by hijacking cellular replication [5]. The ensuing expression of oncogenes and integration of the viral DNA in the genome of infected cells may cause a progressive neoplastic modification of the epithelial tissue [6], called a precancerous lesion. In most cases, infections clear spontaneously thanks to a successful immune response, but in almost 10% of cases [7], [8] HPV eludes the host’s defenses and results in precancerous lesions, which can progress over years to more severe forms and eventually to cancer. Two HR HPV genotypes (16 and 18] are responsible alone for almost 80% of all cervical cancers worldwide [9].

Two different vaccines are available for the prevention of HPV-related cancers, both targeting HPV 16 and 18. The vaccines have shown an efficacy close to 100% in preventing precancerous lesions in HPV-naïve individuals [10], [11], without significant evidence for waning immunity throughout the duration of the trials [12], [13]. Protection against precancerous lesions is also conferred to HPV-experienced individuals (cohort termed as “intention to treat”, ITT, in the clinical trials), but with a lower efficacy than for the naïve (cohort termed as “adherent to protocol”, ATP) [10], [11]. The two vaccines did not have a significant effect on the rates of regression or progression of precancerous lesions in women with neoplasias of any grade at the moment of vaccination [10], [14]. These vaccines properties, coupled with the rapid acquisition of HPV infection at ages following sexual debut [15] have induced authorities in many countries to consider the female adolescents in the pre-sexual age as primary target of the vaccination [16].

The widespread diffusion of HPV infection puts all female sexually active population at risk of cancer. For this reason in the past decades, many countries worldwide have initiated cervical screening programs, aimed at the timely detection and treatment of precancerous lesions [17]. In Italy, a screening program targeting women between 25 and 70 years has been in place since 1996, allowing a sharp reduction in cancer incidence [18]. Vaccination programs for young females (12-year-old) against HPV were also initiated in 2008 reaching different coverages on a regional basis [19].

In this work, a mathematical model is used to evaluate the effect of adding an immunization program for females aged 12 years against HPV 16 and 18 only (i.e. without considering the possible effects of cross-immunization versus other HR HPV genotypes) to the existing screening protocol. Vaccination schedules combining pre-adolescents immunization with various catch-up options at different ages are also evaluated with respect to the predicted incidence of pre-cancerous lesions and cervical cancers and to the number of needed treatments.

## Materials and Methods

### Data

Compared to previous modeling studies on HPV in Italy [20]–[22] we had access to new datasets about two critical aspects: sexual behavior and HPV prevalence. On the former, we combined data from two recent surveys, i.e. the national Survey of Italian Sexuality [23], and a large scale survey on individuals below 25 years in 4 Italian regions [24]. On the latter, age-specific prevalence of HR-HPV infections in Italian women were retrieved from three large scale studies [25]–[27], selected on the basis of sample size, coverage of territory and age groups, and complementarity about these dimensions (Table 1). These data were pooled together to obtain the dataset of HPV prevalence in women used for model parametrization. The age-specific contribution of different HR types reported in two of the selected studies [26], [27] was used to extract the contribution of infections from vaccine-covered types (HPV 16 and 18). Age-specific incidence of cervical cancer was provided by the Itacan database of the Italian Cancer Register (AIRTUM) [28]. These figures were weighted by the age-specific proportions of cancers attributed to HPV types 16 and 18, reported in three large Italian studies [29]–[31].

**Table 1:** Reference studies for HPV prevalence data and study details.

Reference for HPV prevalence data	Sample size	Age-range	Area
NTCCC [25]	49,841	26+	Northern & Central Italy
Giorgi Rossi et al, 2010 [26]	3,817	26+	Central & Southern Italy
PreGio [27]	2000	18–26	Italy

Screening coverages for 1996–2008 were provided by the National Observatory on Screening [18]. Vaccination coverages for 2008–2012 were obtained from the Italian Public Health Institute [19]. Demographic data (yearly births and age-specific mortality rates) were provided by the Italian National Institute of Statistics [32].

### Mathematical Model

A mathematical model described by ordinary differential equations was built to reproduce the heterosexual transmission dynamics of HPV infections caused by types 16 and 18, and progression from infection through various stages of disease. The population is assumed to be at demographic equilibrium with a fixed inflow of births per year and a realistic but time-invariant life table, and is structured by sex, chronological age, and sexual activity level, as standard for HPV models [33]. In particular we consider 100 one-year age classes with constant rate transitions between age groups, and three sexual activity levels.

**Figure 1:**
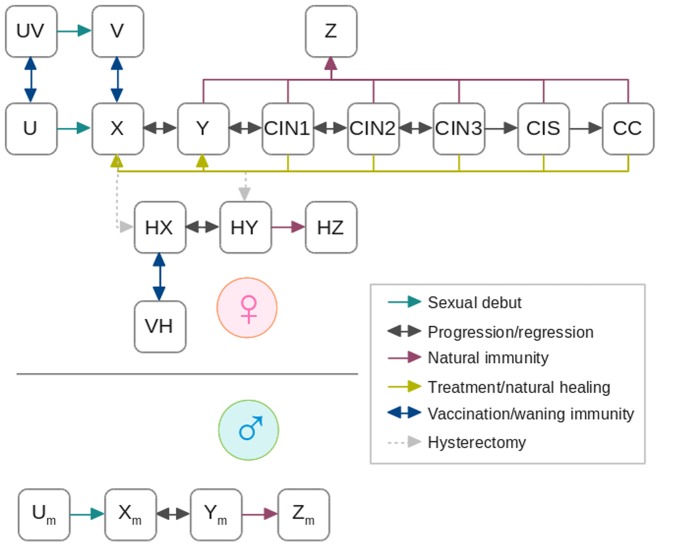
A simplified flowchart of the compartmental model including the main compartments and transitions. The scheme is stratified by 100 one-year age-classes and replicated by 3 sexual activity levels. U: sexually inactive women; UV: sexually inactive, vaccinated women; X: sexually active, susceptible women; Y: women with HPV infection; CIN1–CIN3: women with cervical intraepithelial neoplasia, grades 1–3; CIS: women with carcinoma in situ; CC: women with cervical cancer; Z: immune women; V: vaccinated women; HX: hysterectomized susceptible women; HY: hysterectomized women with HPV infection; HZ: hysterectomized immune women; VH: vaccinated, hysterectomized women; U_m_: sexually inactive men; X_m_: sexually active, susceptible men; Y_m_: men with HPV infection; Z_m_: immune men.

A flow-diagram of the model for a specific age class and sexual activity level is reported in Figure 1. Newborns are assumed to enter the sexually inactive compartment (U), until they experience their sexual debut, thereby becoming sexually active and susceptible to HPV infection (X). Contrary to most available models, which assume a fixed age at sexual debut, we explicitly model the transition to the sexually active phase by an age-dependent rate specific for each sex, fitted to sexual debut data with a Hernes-type model [34] (see Materials S1 for details).

Susceptible individuals of both sexes acquire HPV infection at a time-varying rate (termed “force of infection”, FOI) specific for sex, age and sexual activity level [33], [35]–[37]. The FOI depends on mixing among classes of individuals, which has both a proportionate and a preferential component [36]. HPV-infected females can clear the infection or develop precancerous lesions (cervical intraepithelial neoplasia, CIN) of progressive gravity (CIN-1–CIN-3) and eventually carcinoma in situ (CIS) and cervical cancer (CC). Individuals with precancerous and cancerous lesions are also capable to transmit the infection and therefore contribute to the FOI. Lesions of all grades also have a grade-specific probability of regressing to the previous grade or of spontaneous healing. Individuals naturally healing from infection move to either the susceptible or the immune compartment according to a probability *z* of developing permanent immunity. Lesions of grade CIN-2 or higher have a severity-specific probability of being diagnosed (either by spontaneous symptoms, or by screening) and treated. The rates of diagnosis and treatment for cervical screening were calculated in such a way to mimic the Italian screening protocol [18]. We assumed that women with lesions that have been treated do not develop immunity and have a probability of retaining infection.

A severity-specific proportion of treated lesions [18] occurs by hysterectomy, which removes the risk of CIN and cancers. Women can also undergo hysterectomy for reasons different from treatment of HPV lesions, at an age-specific rate [38]. Hysterectomized women may acquire HPV infections, transmit, heal and develop immunity at the same rates as the general population [40].

Full technical details on the model are given in Materials S1.

### Preventive Strategies

HPV vaccination is included through a flexible schedule considering both routine immunization for females aged 12 years and catch-up immunization. Vaccination is assumed to be effective only on individuals not currently infected with HPV, and its protective effect can wane in time. We first consider three hypothetical reference scenarios: i) “no-intervention”, where neither screening nor vaccination are considered; ii) “screening only”, where the screening program implemented during 1996–2008 is continued thereafter at the 2008 level (coverage at about 60%); iii) interrupted “actual vaccination”, considering, in addition to the continuation of current screening program, vaccination of females aged 12 years only during 2008–2012, at vaccine uptake observed in Italian regions since 2008 [19]. In addition to the three reference scenarios, we consider two further immunization scenarios: iv) “baseline”, where only 12-year-old girls are routinely vaccinated from 2013; v) “catch-up”, where “baseline” is augmented with a catch-up program targeting 25-year-old women until 2022, i.e. until the first cohort of routine vaccinees (born in 1997) has reached the catch-up age. Different ages of catch up (namely at 14, 16, 18, 20 and 22 years) are also considered for comparison. In all cases, we assumed a vaccine efficacy of 95% and an average duration of protection of 50 years. The expected coverage for both routine and catch-up programs are set to 70%, consistently with the average coverage of the regional programs up to date [19]. We consider a simulation horizon of 100 years starting in 2013. A univariate sensitivity analysis with respect to these three parameters has also been performed. Minimum and maximum values of efficacy are taken from the confidence interval estimated during clinical trials [10], [11]; minimum value for duration is taken from a mathematical model of antibody decay from the same clinical trials [39].

### Model Parametrization

As most available models of HPV transmission dynamics [21], [33], [37], [41], [42], our model has a large number (K = 16) of unknown parameters. The large dimension of the parameter space, the possible correlation between parameter effects and the paucity of appropriate data make accurate identification unfeasible. The model parametrization was therefore carried out in two steps: first, we estimated parameters related to natural history (NH) of infection (KNH = 6, see Table 2) by fitting the age-specific prevalence of HPV types 16 and 18 predicted by the model to the corresponding observed prevalence [25]–[27]. Second, based on the estimates of natural history parameters, we estimated parameters related to progression and regression (PR) of cervical lesions (KPR = 10, see Table 2) from the age specific incidence of cancer in women below 60 years during 2004–2008 [28]. This approach is justified by the negligible contribution of lesions and cancers to the overall HPV prevalence [25] and therefore to the FOI.

**Table 2:** Best estimates of model parameters.

Symbol	Description	Unit	Optimal
Natural history parameters			
β_MF_	Probability of male to female infection per partner	%	92.6
β_FM_	Probability of female to male infection per partner	%	61.8
ε_a_	Coefficient of assortativity by age	–	0.902
ε_l_	Coefficient of assortativity by sexual activity level	–	0.995
z	Probability of acquiring natural immunity	%	17.9
d	Average duration of lesion-free infections	yr	2.00
Progression/regression parameters			
H_Y1_	Rate of progression from infection to CIN1	yr^−1^	0.098
H_1X_	Rate of clearance of CIN1	yr^−1^	2.14
H_12_	Rate of progression from CIN1 to CIN2	yr^−1^	0.186
H_2X_	Rate of clearance of CIN2	yr^−1^	0.423
H_21_	Rate of regression from CIN2 to CIN1	yr^−1^	0.260
H_23_	Rate of progression from CIN2 to CIN3	yr^−1^	0.465
H_3X_	Rate of clearance of CIN3	yr^−1^	0.010
H_32_	Rate of regression from CIN3 to CIN2	yr^−1^	0.038
H_3CIS_	Rate of progression from CIN3 to CIS	yr^−1^	0.060
H_CIS-CC_	Rate of progression from CIS to CC	yr^−1^	0.028

CIN: cervical intraepithelial neoplasia; CIS: carcinoma in situ; CC: cervical cancer.

Model predictions of age-specific prevalence of infection (considering in this category all women with a simple HPV infection or with CIN at any stage of progression or with cervical cancer) and incidence of cervical cancer were calculated by running the model until equilibrium and then introducing the screening program (at a simulation time corresponding to 1996, the year of initiation of the program) with a time-varying coverage following official estimates [18]. Since these estimates were given only until 2008, we assumed a constant coverage equal to that of 2008 for the following years.

Parameter estimates were computed by exploring the whole parameter space by Latin Hypercube Sampling (LHS) [43], using M = 10000 parameter constellations. Values of each parameter were drawn from uniform distributions whose plausible ranges were assigned by a broad literature search (see Materials S1). In the first step of the parametrization, we calculated the Poisson likelihood of the age-specific HPV prevalence predicted by the model for each parameter constellation. We then selected the minimum and maximum values of natural history parameters within the top 5-percentile of the likelihood score and used these values to redefine the range of exploration for the natural history parameters. In the second step the LHS sampling was repeated using the updated ranges for natural history parameters, so that the new search was localized to a region of the parameter space that predicted an age-specific HPV prevalence curve compatible with observations. Model predictions obtained with the newly sampled parameters were compared with the observed age-profile of both HPV prevalence and cancer incidence. This time, the goodness of fit was measured by the root mean squared relative error E_R_:
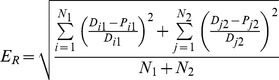



N_1_ is the number of data points for HPV prevalence and D_▪1_ and P_▪1_ are the vector of observations and model predictions, respectively. Similarly, N_2_ is the number of data points for cervical cancer incidence and D_▪2_ and P_▪2_ are the vector of observations and model predictions. This error allows goodness of fit evaluations for heterogeneous data having different order of magnitudes, as is the case for HPV infection prevalence and cancer incidence [44]. To improve the fit, the second step was further iterated, after restricting with the same criterion the range of all model parameters (rather than only those related to the natural history). This additional sub-step was made necessary by the large uncertainty on the range of progression and regression parameters and by the strong correlation of their effects. At the end of the fitting procedure, the optimal parameter set corresponding to the minimum E_R_ (reported in Table 2) was selected as the best estimate of parameter values and used throughout the rest of this study.

The robustness of model predictions with respect to alternative best-fitting parameter sets is assessed in Materials S1.

## Results

**Figure 2:**
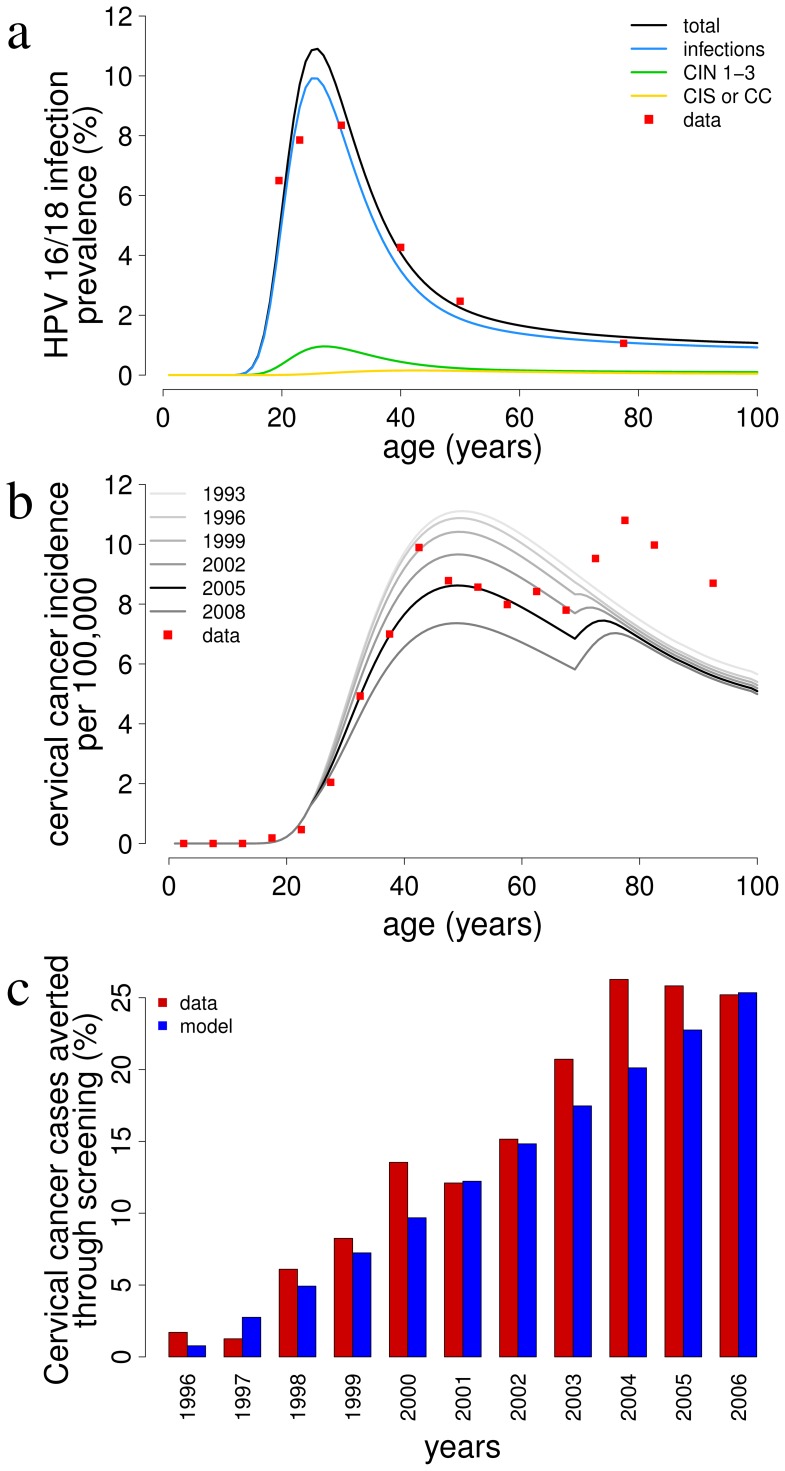
Results of model fitting. a) prevalence of HPV 16/18 in Italian women [25]–[27] by age groups and corresponding curve predicted by the model, disaggregated by infection type; b) cervical cancer incidence data by age due to HPV 16 and 18 [28]–[31] and as predicted by the model over time. Data refer to the period 2004–2006 and need to be compared with the 2005 curve (darkest line in Figure). Note the change in shape with the appearance of a second peak at ages >70 years after the introduction of screening in 1996, consistently with observations in other countries [45]–[46]; c) comparison between observed [28] and predicted screening effectiveness over time in terms of percent reduction in number of cases with respect to the baseline value of 1996.

Figure 2 reports the comparison between the model fit and corresponding data. Figure 2a shows that the model captures the age specific prevalence of HPV 16/18 infections (including any stage of progression to precancerous lesions or cancer) recently observed in Italian women [25]–[27], predicting a peak of prevalence of 10.9% at age 26. Figure 2a also shows that, according to the model, the majority of prevalent infections is lesion-free, consistently with the low prevalence of all-HPV-types lesions found in Italian women [25].

In Figure 2b the age-specific profile of cervical cancer incidence in Italian women ([28], average over years 2004, 2005 and 2006) is shown, together with the predicted age-specific incidence of cervical cancer at time intervals of three years, starting from the model’s steady state (in 1993) and up to 2008. Observed data show a bimodal shape, with a first peak in the 40–45 years age group, and a second, higher peak in the 75–80 years age group (Figure 2b, red squares). As shown by the analysis of historical time series on age-specific cervical incidence in European countries [45], [46], bimodality in cancer incidence is a consequence of the initiation of a screening program covering the population only up to a given age (i.e. 70 years in Italy). In particular the peak at high ages arises, and is gradually magnified over time, by the sudden increase of the population at risk of cervical cancer which occurs at the exit of the screening age, contrasted with the cumulative success over time of diagnosis and treatment within the screened age groups [45], [46]. The incidence curve predicted by our model in 2005 shows a very accurate quantitative reproduction of cancer data until age 70, and it is capable to at least qualitatively reproduce the second mode appearing after age 70. The ability to reproduce the bimodality is a predictive feature of our model, since no specific information on the shape of the age-specific incidence was used, either in model design or during parametrization (indeed, only cervical cancer incidence up to 60 years of age is used). Bimodality in model predictions progressively emerges just after the introduction of the cervical screening programme in Italy in 1996 (Figure 2b), consistently with epidemiological findings [45], [46]. For women under 70 years of age, there is a strong quantitative agreement between model predictions relative to 2005 and the data. In older women, predictions become quantitatively inaccurate. This relative lack of accuracy can be explained by two factors. First, cancers in older women derive mostly from infections transmitted decades before. The equilibrium approximation of the model, therefore, becomes increasingly inaccurate at these ages because of possible historical changes in structural factors (e.g. sexual behavior, population profiles of immunity, etc.). Second, in age groups after the exit of the screening program the average delay between cancer and diagnosis increases suddenly, because the whole population returns unscreened, thereby creating a bulge in cancer diagnoses a few years later. However, the model is still able to correctly capture the age at the second peak of cancer incidence found in data.

As a further model validation, Figure 2c shows the percentage reduction in incident cervical cancer cases with respect to pre-screening levels (incident cases in 1995) according to data [28] and the model. A very good agreement is shown, with an average yearly reduction of 2.3%, both in the data and in the model.

Given the good agreement with data, the model was used to predict the likely effects of different control scenarios on the incidence of cervical cancers due to HPV 16/18 in Italy.

**Figure 3:**
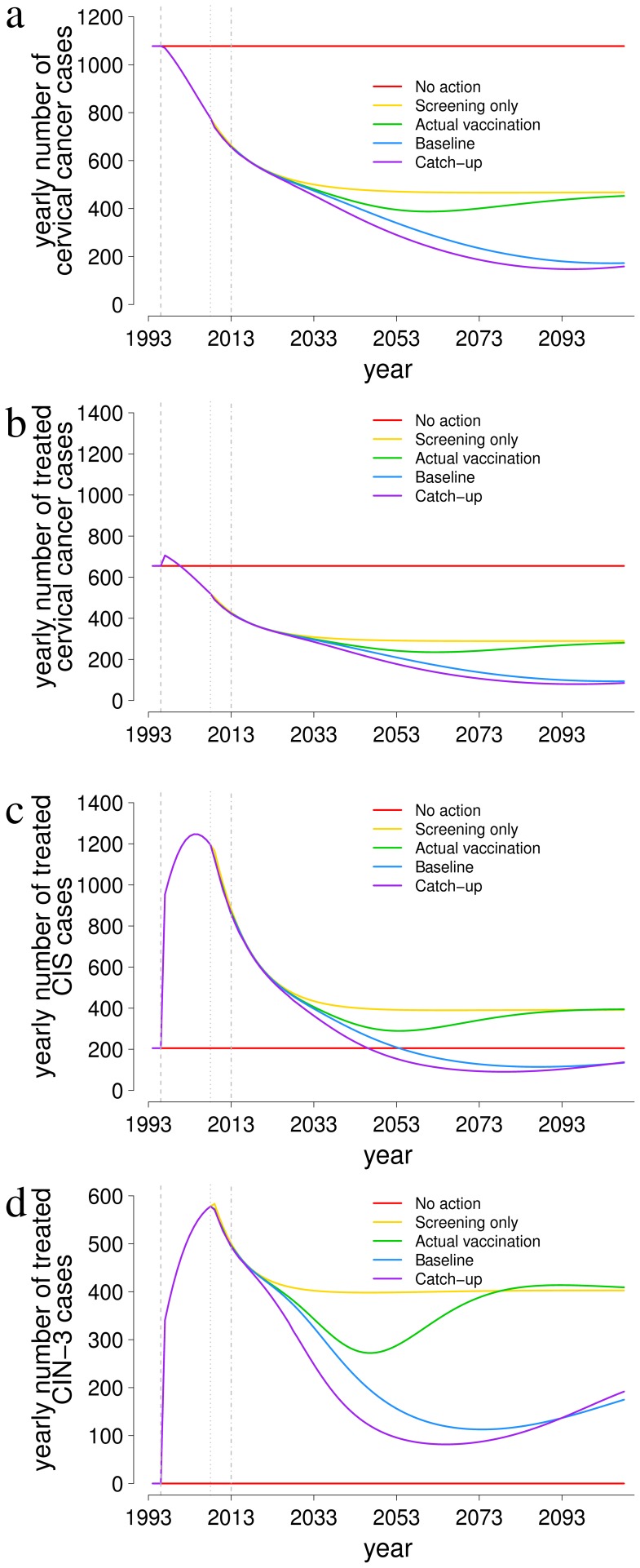
Evaluation of different prevention strategies. Predicted number of cervical cancer cases (a) and treatments of cervical cancer (b), carcinoma in situ (CIS) (c) and cervical intraepithelial neoplasia (CIN) grade 3 (d) over time under different prevention strategies are compared in this figure. *No action*: model equilibrium, in the absence of both screening and vaccination; *screening only*: screening with realistic effective coverage until 2008, and then kept constant coverage from 2009; *actual vaccination*: as screening only, with the addition of the implemented program of immunization of 12-years-old girls in 2008–2012, with realistic coverage, assumed to be discontinued from 2013 on; *baseline*: as actual vaccination, but the vaccination program is assumed to continue indefinitely with coverage equal to 2012; *catch-up*: as baseline, including a catch-up program for 25 year-old women.

Figure 3a shows the projected number of incident cervical cancer cases due to HPV 16/18 in the five scenarios defined in section Methods. According to model and data, the screening program (“screening only” curve) reduced cancer incidence by about 40% between 1993 and 2013. The beneficial effects of screening are predicted to extend in the next decades, with a further 25% reduction relative to the current incidence expected in the next 20 years, provided the program is not modified. The vaccination program in 2008–2012 only (“actual vaccination”) has hardly had any impact yet, but might yield a transitory 8.3% reduction in cancer incidence with respect to the “screening only” scenario, corresponding to almost 4,000 cases averted overall prior to return to the “screening only” equilibrium. In the “baseline” scenario the cumulative number of cancer cases and the projected yearly incidence at equilibrium will greatly decrease with respect to the screening-only scenario (−33% and −63% respectively). Finally, catch-up vaccination of 25-year-old women will yield an additional reduction in the cumulative number of incident cases of 9.6% (about 3,000 cases) with respect to the routine vaccination only.

Figures 3b–d show the number of treated cases of cervical cancer, CIS and CIN-3 lesions over time for all scenarios considered. The “no intervention” line represents cases which are treated due to spontaneous care-seeking by patients upon presentation of symptoms. As expected, the active detection of cervical lesions through screening increases the number of treatments in the first few years after its introduction. However, the reduction in cancer incidence allowed by screening reduces the total number of yearly treated cases to levels lower than the pre-screening era in about 5 years (Fig. 3b). The qualitative relation between the effectiveness of different scenarios in reducing the number of treatments holds for all three types of lesions.

**Figure 4:**
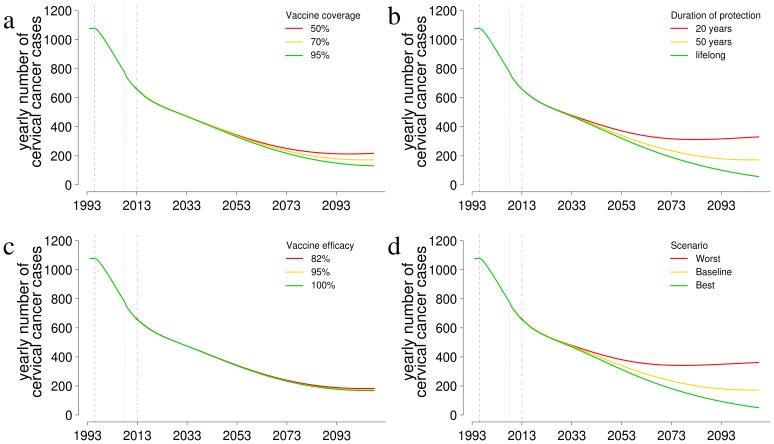
Sensitivity analysis of model predictions with respect to different assumptions on vaccine parameter values. a) vaccine coverage; b) duration of protection; c) vaccine efficacy; d) sensitivity of model predictions when considering the worst and best case of the three parameters together.

Figure 4 shows a sensitivity analysis of the predicted incidence of cervical cancers with respect to assumptions on coverage (Figure 4a), duration of vaccine protection (4b) and vaccine efficacy (4c). Figure 4d considers the best-case and worst-case scenarios where coverage, duration and efficacy are all assumed to be at the highest and lowest values respectively. Figure 4 shows that duration of protection is the most critical parameter and accounts for much of the expected variability between the best-case and worst-case scenarios. In the best case scenario a 13% reduction in cumulative cancer cases and a 70% reduction in yearly incidence is expected with respect to the baseline. The reduction of the best case with respect to the worst case scenario is 30% for the cumulative cancer cases and 86% for the yearly incidence.

**Figure 5:**
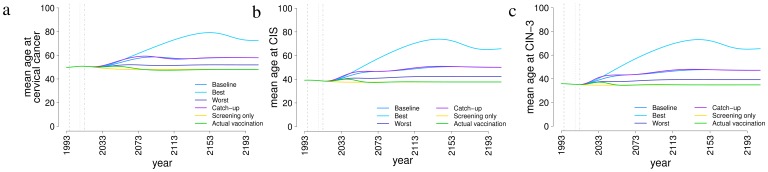
Impact of different prevention strategies on age at onset of severe lesions. Mean age at onset of cervical cancer (a), carcinoma in situ (CIS) (b) and cervical intraepithelial neoplasia (CIN) grade 3 (c) over time, under different prevention strategies. *Screening only*: screening with realistic effective coverage until 2008, and then kept constant coverage from 2009; *actual vaccination*: as screening only, with the addition of the implemented program of immunization of 12-years-old girls in 2008–2012, with realistic coverage, assumed to be discontinued from 2013 on; *baseline*: as actual vaccination, but the vaccination program is assumed to continue indefinitely with coverage equal to 2012; *catch-up*: as baseline, including a catch-up program for 25 year-old women; *best*: as baseline, but with best-case vaccine parameters (coverage: 95%; efficacy: 100%; duration of protection: permanent); *worst*: as baseline, but with worst-case vaccine parameters (coverage: 50%; efficacy: 82%; duration of protection: 20 years).

Figure 5 shows the effect of different vaccination scenarios on the mean age at cervical cancer, CIS and CIN-3 lesions respectively. Figure 5a shows that the current mean age at cervical cancer is around 50 years, and will decrease by about two years in the screening-only and actual vaccination scenarios, i.e. those not envisioning a long-term vaccination program. This reduction is due to a more consistent reduction introduced by the screening program in more advanced ages, where cervical cancer is more frequent. The average age at cervical cancer at equilibrium will be delayed by about 7 years in the baseline and in the catch-up scenarios, by 2 years in the worst case scenario and by 22 years in the best case scenario. Similar considerations can be done for the mean age at CIS and CIN-3 (Figures 5b and 5c).

**Figure 6:**
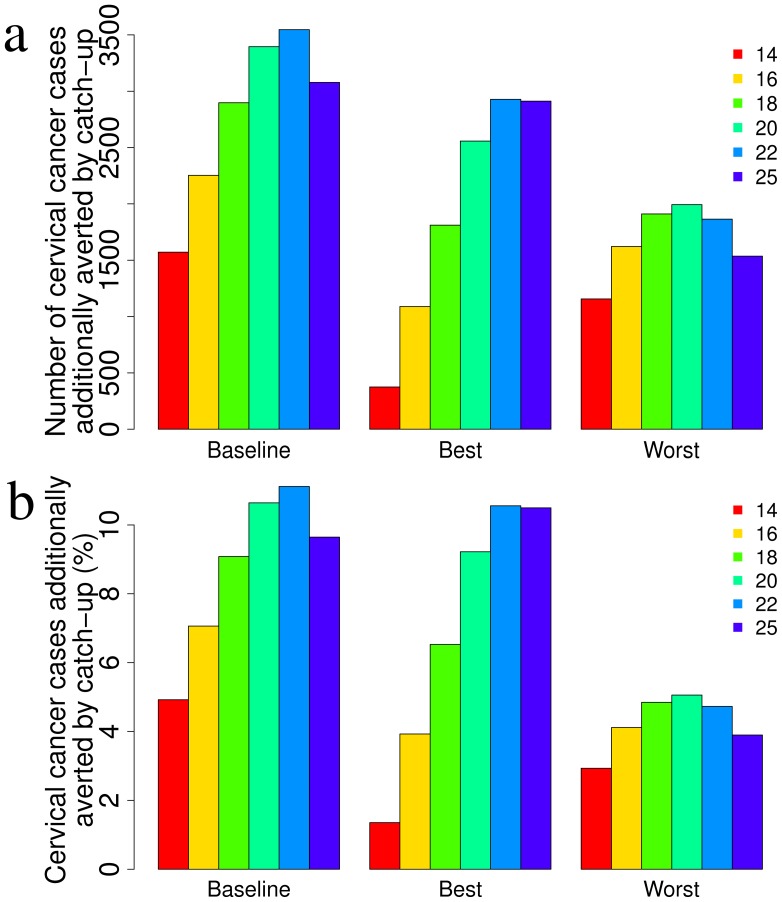
Efficacy of catch-up strategies administered at different ages. Efficacy is represented in terms of number (a) and percentage (b) of additionally averted cervical cancer cases with respect to the baseline scenario of immunization at 12 year only. In each panel, a sensitivity analysis of the baseline scenario with respect to the best and worst case is represented.

Figure 6 compares the efficacy of targeting different ages in the catch-up program with respect to the baseline, worst-case and best-case vaccination scenario. Figure 6a reports the number of cumulative cervical cancer cases additionally averted through catch-up with respect to the corresponding scenario without catch-up. Figure 6b shows the same figures in terms of percent reduction. Overall, the optimal effectiveness of the catch-up program occurs for the baseline scenario. Indeed, in the best-case scenario, the reduction in incidence due to routine vaccination is so high that few additional cases can be avoided by catch-up; whereas in the worst-case scenario the vaccine is not sufficiently effective and long lasting to prevent a consistent additional number of cancers with catch-up. In all scenarios, the optimal age for catch-up vaccination is between 20 and 22 years.

## Discussion

This study uses a model of heterosexual HPV transmission and development of cervical lesions and cancer to provide perspectives on the impact of HPV female immunization in Italy. The model presents a necessarily simplified representation of the complex epidemiology of HPV, whose pathogenesis and immune response are still poorly understood [47]. One of the most controversial issues regards the mechanism of development of natural immunity [48]. Although several large-scale studies were conducted [49]–[53], results are ambiguous, ranging from no effect of previous infection [49] to 64% reduction in re-infection risk [50]. Reconciliation of these contradictions is, at the current moment, speculative [48]. Another key source of uncertainty are the ecological interactions between different HPV genotypes. A certain degree of cross-immunity between HPV types has been observed [9], and the compatibility of this hypothesis with pre-vaccination epidemiological data has been recently shown [54]. However, the complex ecological dynamics among different HPV types and the theoretical possibility of partial ecological replacement in a context of realistic vaccination programs have not yet been studied using mathematical models. Models that consider multiple HPV types for vaccination studies generally assume the simple case of ecological independence, i.e. no ecological interaction among types (e.g. [42]). Our work implicitly assumes that immunity against either type 16 or 18 provides complete cross-protection against the other, as in other works [33], [37]. This assumption has been shown to result in conservative estimates of vaccine effectiveness with respect to the assumption of ecological independence [55]. Additionally, the current model structure, including heterosexual transmission only, is a simplification due to data paucity. Indeed 56% of men having sex with men have been found to be infected with HR HPV types in Italy [56] and therefore they could represent a core group [57] for transmission to females through bisexual activity [58]. Thus a more comprehensive representation of sexual behavior might be important for reproducing the natural history of HPV and better evaluating the impact of vaccination.

Two main characteristics distinguish our model from previously published ones. First, instead of assuming a fixed age at sexual debut, we consider a realistic age-specific rate of entrance in the sexual activity classes. This may be important to better capture the age patterns at which HPV infection is acquired for the first time. Second, we acknowledged the importance of hysterectomy on the age-specific incidence of cervical cancers, which is often underestimated in epidemiological studies [59]. Other mathematical models consider hysterectomized women (e.g. [37]), but they are generally removed from transmission, which is inconsistent with empirical observations [39].

Only one other transmission dynamic model was developed for the Italian context [20] and considers HPV type 16 only. This model proposes an SIR structure with age-dependent progression parameters, and was later applied to considerations on HPV vaccination [21], also comparing results with an SIS version of the same model. The age-specific prevalence data used for model calibration came from women older than 25 years, i.e. in the monotonically decreasing region. We believe that the new data collected in the PreGio study [27] on HPV prevalence in younger ages and used in the present study allow a more precise identification of model structure and therefore a more appropriate description of natural history. Indeed, according to our preliminary simulations, neither the SIR nor the SIS structure alone are able to capture the features of the HPV prevalence curve once data for women younger than 25 years are considered. Four pharmaco-economic studies assessed the effectiveness of HPV vaccination in Italy using static models [22], [60]–[62]. All of them predicted a drastic reduction of cancer incidence at equilibrium. None of them, however, could evaluate the impact of catch-up strategies due to the absence of transmission dynamics.

This work aims to provide a useful contribution to the discussion on HPV immunization in Italy. Our results are consistent with the available modeling literature [22], [63] in suggesting that, provided the screening program continues, vaccinating girls at pre-sexual age (12 years) will be highly effective in preventing a further large number of cervical lesions and cancers in the next decades, and that the largest source of uncertainty in predicting the number of averted diseases is due to the actual duration of vaccine immunity. Results of longitudinal studies [12], [13] and mathematical models [40], [64] suggest that a very long duration of protection should be expected. As for the effectiveness of additional catch-up programmes, the model suggests an optimal catch-up age at around 20–22 years, but a significant number of averted cases is also to be expected if the catch-up vaccination is administered at first entrance in the screening programme, at the age of 25 [24]. Finally, our simulations indicate that, under the best case scenario, female immunization might also result in large delays at onset of CIN3, CIS or cancer, and consequently in a serious improvement in the quality of life of women.

## Supporting Information

Materials S1 **Additional information on model equations, model parametrization and robustness of model predictions with respect to parameter estimates.**
(PDF)
