# Supplementary material for: The Impact of HPV Female Immunization in Italy: Model Based Predictions
Source: PLoS One. 2014 Mar 11;9(3):e91698. doi: 10.1371/journal.pone.0091698 (PMC3950270; doi:10.1371/journal.pone.0091698)
Supplement: Materials S1 — Additional information on model equations, model parametrization and robustness of model predictions with respect to parameter estimates. (PDF) [file pone.0091698.s001.pdf]

The impact of HPV female immunization in Italy: model based predictions

Giorgio Guzzetta[1,2], Luca Faustini[3], Donatella Panatto[4], Roberto Gasparini[4], Piero Manfredi[5].

[1] Trento Rise, Via Sommarive 18, 38123 Povo (Trento) – ITALY
[2] Fondazione Bruno Kessler, Via Sommarive 18, 38123 (Trento) - ITALY
[3] Istat Toscana, Lungarno Colombo 54, 50136 Florence – ITALY
[4] Dipartimento di Scienze della Salute, Università di Genova, Via Pastore 1, 16132 Genoa – ITALY
[5] Dipartimento di Economia e Management, Università di Pisa, Via Ridolfi 10, 56124 Pisa – ITALY

Materials S1

S1. Model details

Full model equations
The model for HPV transmission dynamics and control by screening and vaccination is structured by 100 age-classes (*a)* and 3 sexual activity levels (SAL, *l*) (1: low activity; 2: intermediate activity; 3: high activity), with 23 ordinary differential equations (ODEs) for each couple (*a*,*l*). The equations are reported hereafter with the following notation. U: sexually inactive individuals; X: susceptible; Y: infectious; Z: removed; prefixes before state variables: H, hysterectomized; V: vaccinated. Subscript M, F specifies the gender. Subscript/superscript *k* defines all possible forms of HPV infection: simple infection (no subscript), Cervical Intraepithelial Neoplasia (CIN) of grades 1-3 (C1, C2, C3), Carcinoma in Situ (CIS), Cervical Cancer (CC). For example $X_M[27,2]$ denotes the number of 27-year-old susceptible males belonging to the intermediate SAL, while $Y_F^{C1}[27,1]$ denotes the number of 27-year-old infective females belonging to the low SAL, who have developed CIN-1. The model does not include further compartments for screened individuals because the screening program is considered in the calculation of treatment rates for women with ongoing cervical neoplasies.

<u>Male compartments:</u>

$$\frac{d\,U_M[a]}{dt} = b_M[a] - w\,U_M[a] + w\,U_M[a-1] - \mu_M[a]\,U_M[a] - \delta_M[a]\,U_M[a] - p_M[a]\,e\,U_M[a] + \omega\,VU_M[a]$$

$$\frac{d\,X_M[a,l]}{dt} = -w\,X_M[a,l] + w\,X_M[a-1,l] - \mu_M[a]X_M[a,l] + \delta_M[a]\,f_M[l]\,U_M[a] - \lambda_{FM}[a,l]X_M[a,l] + \gamma\,(1-z)Y_M[a,l] - p_M[a]\,e\,X_M[a] + \omega\,V_M[a]$$

$$\frac{d\,Y_M[a,l]}{dt} = -w\,Y_M[a,l] + w\,Y_M[a-1,l] - \mu_M[a]\,Y_M[a,l] + \lambda_{FM}[a,l]X_M[a,l] - \gamma\,Y_M[a,l]$$

$$\frac{d\,Z_M[a,l]}{dt} = -w\,Z_M[a,l] + w\,Z_M[a-1,l] - \mu_M[a]\,Z_M[a,l] + \gamma\,z\,Y_M[a,l]$$

<u>Female compartments:</u>

$$\frac{d\,U_F[a]}{dt} = b_F[a] - w\,U_F[a] + w\,U_F[a-1] - \mu_F[a]\,U_F[a] - \delta_F[a]\,U_F[a] - p_F[a]\,e\,U_F[a] + \omega\,VU_F[a] - \eta[a]U_F[a]$$

$$\frac{d\,X_F[a,l]}{dt} = -w\,X_F[a,l] + w\,X_F[a-1,l] - \mu_F[a]X_F[a,l] + \delta_F[a]\,f_F[l]\,U_F[a] - \lambda_{MF}[a,l]X_F[a,l] + \gamma\,(1-z)Y_F[a,l] + q\,H_{1X}\,(1-z)Y_F^{C1}[a,l] + q\,H_{2X}\,(1-z)Y_F^{C2}[a,l] + q\,H_{3X}\,(1-z)Y_F^{C3}[a,l] - p_F[a]\,e\,X_F[a] + \omega\,V_F[a] - \eta[a]X_F(a,l) + s\sum_k T_k Y_F^k[a,l](1-h_k)$$

$$\frac{d\,Y_F[a,l]}{dt} = -w\,Y_F[a,l] + w\,Y_F[a-1,l] - \mu_F[a]Y_F[a,l] + \lambda_{MF}[a,l]X_F[a,l] - \gamma\,Y_F[a,l] - H_{Y1}Y_F[a,l] + (1-q)H_{1X}\,(1-z)Y_F^{C1}[a,l] + (1-q)H_{2X}\,(1-z)Y_F^{C2}[a,l] + (1-q)\,H_{3X}\,(1-z)Y_F^{C3}[a,l] - \eta[a]Y_F[a,l] + (1-s)\sum_k T_k Y_F^k[a,l](1-h_k)$$

$$\frac{d\,Y_F^{C1}[a,l]}{dt} = -w\,Y_F^{C1}[a,l] + w\,Y_F^{C1}[a-1,l] - \mu_F[a]Y_F^{C1}[a,l] + H_{Y1}Y_F[a,l] - H_{1X}Y_F^{C1}[a,l] - H_{12}Y_F^{C1}[a,l] + H_{21}Y_F^{C2}[a,l] - \eta[a]Y_F^{C1}[a,l] + q(H_{1X}Y_F^{C1}[a,l] + H_{2X}Y_F^{C2}[a,l] + H_{3X}Y_F^{C3}[a,l])(1-z) - T_{C1}Y_F^{C1}[a,l]$$

$$\frac{d\,Y_F^{C2}[a,l]}{dt} = -w\,Y_F^{C2}[a,l] + w\,Y_F^{C2}[a-1,l] - \mu_F[a]\,Y_F^{C2}[a,l] + H_{12}Y_F^{C1}[a,l] - H_{2X}Y_F^{C2}[a,l] - H_{21}Y_F^{C2}[a,l] - H_{23}Y_F^{C2}[a,l] + H_{32}Y_F^{C3}[a,l] - \eta[a]Y_F^{C2}[a,l] - T_{C2}Y_F^{C2}[a,l]$$

$$\frac{d\,Y_F^{C3}[a,l]}{dt} = -w\,Y_F^{C3}[a,l] + w\,Y_F^{C3}[a-1,l] - \mu_F[a]\,Y_F^{C3}[a,l] + H_{23}Y_F^{C2}[a,l] - H_{3X}Y_F^{C3}[a,l] - H_{32}Y_F^{C3}[a,l] - H_{3CIS}Y_F^{C3}[a,l] - \eta[a]Y_F^{C3}[a,l] - T_{C3}Y_F^{C3}[a,l]$$

$$\frac{d\,Y_F^{CIS}[a,l]}{dt} = -w\,Y_F^{CIS}[a,l] + w\,Y_F^{CIS}[a-1,l] - \mu_F[a]\,Y_F^{CIS}[a,l] + H_{3CIS}Y_F^{C3}[a,l] - H_{CIS-CC}Y_F^{CIS}[a,l] - \eta[a]Y_F^{CIS}[a,l] - T_{CIS}Y_F^{CIS}[a,l]$$

$$\frac{d\,Y_F^{CC}[a,l]}{dt} = -w\,Y_F^{CC}[a,l] + w\,Y_F^{CC}[a-1,l] - \mu_F[a]\,Y_F^{CC}[a,l] - \mu_{CC}[a]\,Y_F^{CC}[a,l] + H_{CIS-CC}Y_F^{CIS}[a,l] - \eta[a]Y_F^{CC}[a,l] - T_{CC}Y_F^{CC}[a,l]$$

$$\frac{d\,Z_F[a,l]}{dt} = -w\,Z_F[a,l] + w\,Z_F[a-1,l] - \mu_F[a]Z_F[a,l] + \gamma z Y_F[a,l] + \sum_k H_{kX}\,z\,Y_F^k[a,l] - \eta[a]Z_F[a,l]$$

<u>*Hysterectomized female compartments:*</u>

$$\frac{d\,HU_F[a]}{dt} = \eta[a]U_F[a] - w\,HU_F[a] + w\,HU_F[a-1] - \mu_F[a]\,HU_F[a] - \delta_F[a]HU_F[a] - p_F[a]\,e\,HU_F[a] + \omega\,VHU_F[a]$$

$$\frac{d\,HX_F[a,l]}{dt} = \eta[a]X_F[a] - w\,HX_F[a,l] + w\,HX_F[a-1,l] - \mu_F[a]HX_F[a,l] + \delta_F[a]\,f_F[l]\,HU_F[a] - \lambda_{MF}[a,l]HX_F[a,l] + \gamma\,(1-z)HY_F[a,l] - p_F[a]\,e\,HX_F[a] + \omega\,VH_F[a] + s\sum_k T_kY_F^k[a,l]h_k$$

$$\frac{d\,HY_F[a,l]}{dt} = \eta[a]\sum_k Y_F^k - w\,HY_F[a,l] + w\,HY_F[a-1,l] - \mu_F[a]\,HY_F[a,l] + \lambda_{MF}[a,l]\,HX_F[a,l] - \gamma\,HY_F[a,l] + (1-s)\sum_k T_kY_F^k[a,l]h_k$$

$$\frac{d\,HZ_F[a,l]}{dt} = \eta[a]Z_F - w\,HZ_F[a,l] + w\,HZ_F[a-1,l] - \mu_F[a]\,HZ_F[a,l] + \gamma\,z\,HY_F[a,l]$$

<u>*Vaccinated compartments:*</u>

$$\frac{d\,VU_M[a]}{dt} = p_M[a]\,e\,U_M[a] - w\,VU_M[a] + w\,VU_M[a-1] - \mu_M[a]\,VU_M[a] - \delta_M[a]VU_M[a] - \omega\,VU_M[a]$$

$$\frac{d\,V_M[a,l]}{dt} = p_M[a]\,e\,X_M[a,l] + \delta_M[a]f_M[l]VU_M[a] - w\,V_M[a,l] + w\,V_M[a-1,l] - \mu_M[a]\,V_M[a,l] - \omega\,V_M[a,l]$$

$$\frac{d\,VU_F[a]}{dt} = p_F[a]\,e\,U_F[a] - w\,VU_F[a] + w\,VU_F[a-1] - \mu_F[a]\,VU_F[a] - \delta_F[a]VU_F[a] - \omega\,VU_F[a] - \eta[a]VU_F[a]$$

$$\frac{d\,V_F[a,l]}{dt} = p_F[a]\,e\,X_F[a,l] + \delta_F[a]f_F[l]VU_F[a] - w\,V_F[a,l] + w\,V_F[a-1,l] - \mu_F[a]\,V_F[a,l] - \omega\,V_F[a,l] - \eta[a]V_F[a,l]$$

$$\frac{d\,VHU_F[a]}{dt} = p_F[a]\,e\,HU_F[a] + \eta[a]VU_F[a] - w\,VHU_F[a] + w\,VU_F[a-1] - \mu_F[a]\,VHU_F[a] - \delta_F[a]VHU_F[a] - \omega\,VHU_F[a]$$

$$\frac{d\,VH_F[a,l]}{dt} = p_F[a]e\,HX_F[a,l] + \eta[a]V_F[a,l] + \delta_F[a]f_F[l]VU_F[a] - w\,V_F[a,l] + w\,V_F[a-1,l] - \mu_F[a]\,V_F[a,l] - \omega\,V_F[a,l]$$

Parameters appearing in the above equations are briefly described in Table S1. Functions $b_M[a]$ and $b_F[a]$, indicating the gender-specific number of newborns per year, are defined in such a way that newborns are actually introduced only in the age class of individuals aged 0:

$$b[a] = \begin{cases} b \; for \; a = 0 \\ 0 \; elsewhere \end{cases}$$

with $b_M = b_F = b/2$ and b the total number of newborns per year.
The age and sexual level-specific forces of infection for each gender are defined as in (S1):

$$\lambda_{FM}[a_M, l_M] = \beta_{FM} \sum_{a_F, l_F} \left(m_M[a_M, l_M, a_F, l_F] \frac{\sum_k Y_F^k[a_F, l_F]}{N_F[a_F, l_F]} \right)$$

$$\lambda_{MF}[a_F, l_F] = \beta_{MF} \sum_{a_M, l_M} (m_F[a_F, a_M, l_F, l_M] \frac{Y_M[a_M, l_M]}{N_M[a_M, l_M]})$$

where *N* represents the total (gender-specific) population of that age and SAL, and *m* are elements of the (gender-specific) sexual contact matrix, specifying how many sexual partnership men (women) of age a_M (a_F) and SAL l_M (l_F) have with women (men) of age a_F (a_M) and SAL l_F (l_M). For further details on the estimation of mixing matrices, see Section S2 of this text.

Table S1: Parameter descriptions

Parameter	Description	Source
b	Gender-specific newborns per year	(S2)
w	Aging rate	(S3)
μ (a)	Gender and age-specific mortality rate	(S2)
δ(a)	Gender and age-specific rate of sexual debut	Estimated from sexual behavior data
f(l)	Gender and SAL-specific proportion	Estimated from sexual behavior data
c(a,a', l, l')	Gender, age and SAL-specific contact rates	Estimated from sexual behavior data
p(a)	Gender and age-specific vaccine coverage	(S4), several scenarios
e	Vaccine efficacy	(S5), Several scenarios
ω	Waning rate of vaccine protection	(S6), Several scenarios
η(a)	Age-specific hysterectomy rate	(S7)
z	Probability of acquiring natural immunity	Free parameter
γ	Rate of clearance of infections	Free parameter
H_{Y1}	Rate of progression from infection to CIN-1	Free parameter
H_{1X}	Rate of clearance of CIN-1	Free parameter
H_{12}	Rate of progression from CIN-1 to CIN-2	Free parameter
H_{2X}	Rate of clearance of CIN-2	Free parameter
H_{21}	Rate of regression from CIN-2 to CIN-1	Free parameter
H_{23}	Rate of progression from CIN-2 to CIN-3	Free parameter
H_{3X}	Rate of clearance of CIN-3	Free parameter
H_{32}	Rate of regression from CIN-3 to CIN-2	Free parameter
H_{3CIS}	Rate of progression from CIN-3 to CIS	Free parameter
H_{CIS-CC}	Rate of progression from CIS to CC	Free parameter
q	Fraction of natural regressions removing infection	(S8)
s	Fraction of treatments removing infection	(S9, S10)
T_{C1}	Treatment rate for CIN-1	Treatment submodel
T_{C2}	Treatment rate for CIN-2	Treatment submodel
T_{C3}	Treatment rate for CIN-3	Treatment submodel
T_{CIS}	Treatment rate for CIS	Treatment submodel
T_{CC}	Treatment rate for CC	Treatment submodel
h_{C1}	Fraction of hysterectomies for CIN-1 treatments	(S11)
h_{C2}	Fraction of hysterectomies for CIN-2 treatments	(S11)
h_{C3}	Fraction of hysterectomies for CIN-3 treatments	(S11)
h_{CIS}	Fraction of hysterectomies for CIS treatments	(S11)
h_{CC}	Fraction of hysterectomies for CC treatments	(S11)
μ_{CC}(a)	Age-specific cancer-induced mortality rate	(S12)

π	Gender-specific transmission probability per-partnership	Free parameter
ε_a	Age-specific coefficient of assortativity	Free parameter
ε_l	SAL-specific coefficient of assortativity	Free parameter

The treatment submodel

Treatment rates T_k, specified in the model for each lesion type, were calculated by the sum of two contributions: spontaneous care-seeking after the insurgence of symptoms, represented by T_k^{sympt} , and active case-finding by screening, T_k^{screen}.

For spontaneous care-seeking, treatment rates were assumed to be 0 for k = CIN-1, CIN-2, CIN-3, since these lesions were assumed to be asymptomatic; for k=CIS, CC, the rates were estimated as the reciprocal of the sum of the average time from lesion initiation to CIS or CC symptom development (S13) and of the average time from care-seeking to treatment (S14).

The current screening protocol in Italy refers to the Bethesda guidelines and can be briefly summarized (with some minor simplification) as follows: screened women undergo a preliminary cytological Papanicolau (Pap) test; in case of Pap positivity, a colposcopic exam with optional biopsy is performed to ascertain the presence and degree of the lesion and, where appropriate, decide for treatment. Therefore, we calculated the screening-related lesion-specific treatment rates as the product of several terms:

- the time-varying effective screening coverage (Table S2), distributed over a period equal to the screening interval (3 years);
- the proportion of cases discovered per screening round, defined as the product of:
    - the lesion-specific sensitivity of cytologic screening (S15-S17);
    - the compliance to follow-up upon discovery of cytological abnormalities (S11);
    - the lesion-specific sensitivities at colposcopy or biopsy (S18; S19)
- the lesion-specific fraction of treated vs. untreated lesions (S11);
- the reciprocal of the waiting time from care-seeking to treatment (S14).

Table S2: effective screening coverage by year (S11)

Year	Coverage (%)
1996	21.8
1997	24.9
1998	28.1
1999	31.2
2000	34.4
2001	37.6
2002	40.7
2003	43.9
2004	47.1
2005	50.2
2006	53.4
2007	56.6
2008	59.7

S2. Modeling and parametrization of sexual activity

Modeling and parametrization of the age at sexual debut

The high speed at which young people acquire HPV infection in Italy and elsewhere suggests that it might be important to explicitly incorporate the process of transition into the sexually active phase (known as sexual debut, SD) as a separate sub-process of the transmission model. In this feature our model differs from most available models of HPV which usually assume a fixed age at SD in correspondence of which everyone becomes sexually active.

In order to parametrize the process of transition to the sexually active phase, a variety of alternative parametric hazard models were fitted to data on age at sexual debut. The models considered included classical hazard models such as Weibull, Gomperz and Gamma models, and other duration models used in socio-demography, such as Hernes-type (S20). Models were fitted by maximum likelihood for censored data to account for the presence in the available data of both right censoring (individuals who could not specify an age at SD because they were not yet sexually active

at the survey time) and interval censoring (due to individuals who were unable to recall the exact date, i.e. the day, of their SD, but could bound it with some prescribed degree of accuracy i.e. either the month, or the season, or eventually the year when sexual debut occurred). A first selection of the candidate models for the hazard of becoming sexually active was carried out on an empirical basis by computing the maximum likelihood estimate, still including censored data, of a piece-wise constant hazard model, and retaining only those models whose hazard at least qualitatively fit the empirical piece-wise constant hazard. This step allowed to exclude Weibull and Gomperz risks as non-relevant due to their ever-increasing shape. Selection among remaining models was based on likelihood-ratio tests. The model eventually selected was Hernes-type briefly described hereafter.

The Hernes' model

Let T be the random variable describing the age at which transition to the sexually active phase occurs. Let moreover *a* denote age (or time), and consider an age-continuous (or time-continuous) hazard process whose survival function, which represents the probability that the event of interest occurs after any given age duration *a*, is denoted as: $p(a) = P\{T > a\}$. The corresponding density and hazard functions are denoted respectively by $f(a) = -p'(a)$ and by $\lambda(a) = -\frac{p'(a)}{p(a)} = \frac{f(a)}{p(a)}$. Hernes' model is based on the following hazard rate function:

$$\boldsymbol{\lambda(a) = -Ke^{-Ha}\big(1 - p(a)\big)}.$$

The previous model therefore represents an SI-type model for infection diffusion in a homogeneously mixing, fixed and demographically stationary population, characterised by an exponentially declining age-dependent transmission rate $\beta(a) = Ke^{-Ha}$. The model can be solved analytically under the initial condition $p(0) = p_0 < 1$ (necessary to initiate transmission as in all "epidemiologically-based" models) yielding the following closed form for the hazard rate and survival functions:

$$p(a) = \frac{p_0}{(1-p_0)e^{\frac{K}{H}(1-e^{-Ha})} + p_0}$$

$$\lambda(a) = \frac{(1-p_0)e^{\frac{K}{H}(1-e^{-Ha})}Ke^{-Ha}}{p_0\left((1-p_0)\,e^{\frac{K}{H}(1-e^{-Ha})} + p_0\right)}$$

Let us denote by $\vartheta = (K, H)$ the vector of unknown parameters of Hernes' model. Let n, m, p respectively denote the overall sample size, the number of observations for which the age at SD was recorded "exactly" (where exactly means that the date of the day of SD was available), and the number of interval-censored observations, for which the age of SD was recorded as the interval $\left(x^*_{j-1} < T_j < x^*_j\right)$. The likelihood function for sexual-debut data has the following form:

$$L(\vartheta) = \prod_{i=1}^{m} f_X(x_i, \vartheta)\left(\prod_{j=1}^{p} P\left(x^*_{j-1} < T_j < x^*_j\right)\right)^{n-(m+p)}\prod_{h=1} P\left(T_h > t_f\right) =$$

$$= \prod_{i=1}^{m} f_X(x_i, \vartheta)\left(\prod_{j=1}^{p} P\left(x^*_{j-1} < T_j < x^*_j\right)\right)^{n-(m+p)}\prod_{h=1} P_X\left(t_f, \vartheta\right) =$$

The likelihood function was maximised numerically by the function *nonlin* of software R. The results of the fit are reported in Figure S1 and S2.

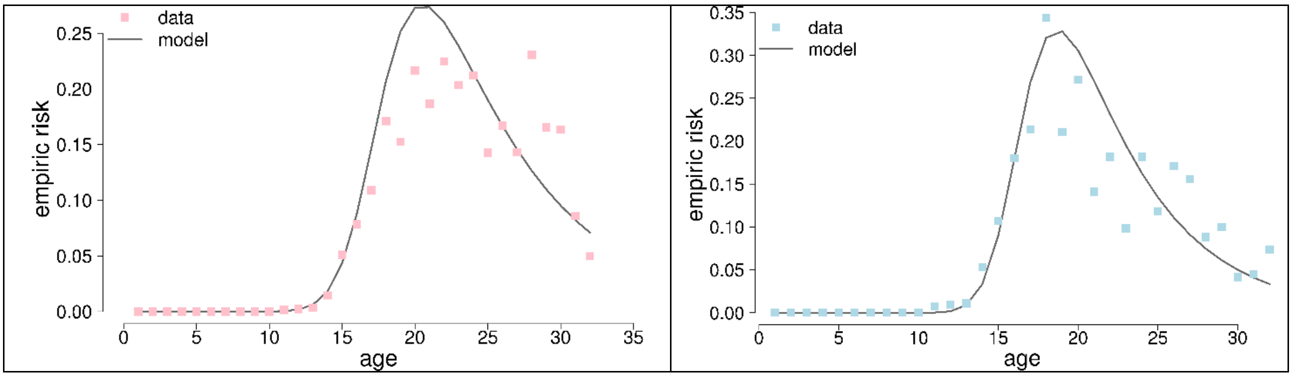

Figure S1. Age at sexual debut in Italy: fit to the empirical risk at SD (computed by a piecewise-constant hazard models for censored data) by the best Hernes type model. Left panel: females; right panel: males.

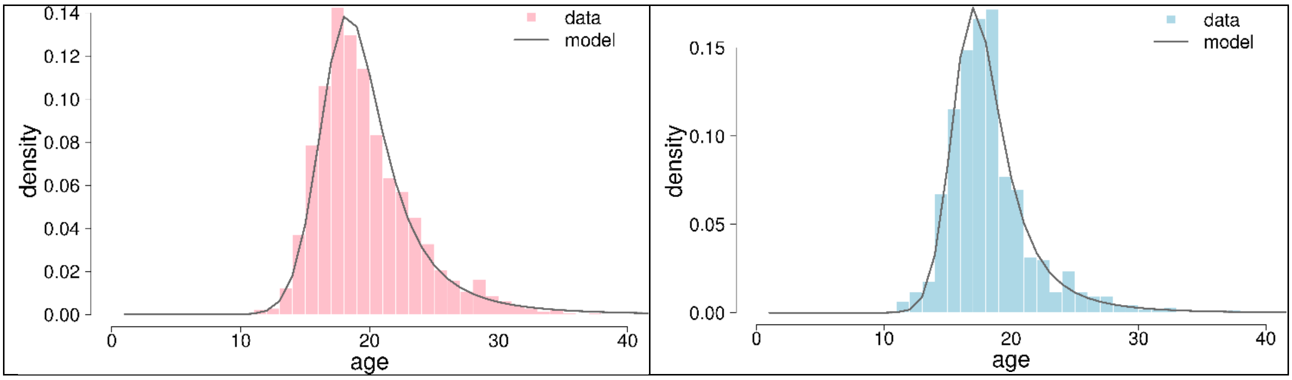

Figure S2. Age at sexual debut in Italy: fit to the observed ages at SD by the best Hernes type model. Left panel: females; right panel: males.

Contact patterns

Considering that our model includes transition to the sexually active population as a separate process, contact patterns were estimated from the subset of sexually active individuals (identified as those who experienced the sexual debut) only, rather than including also non-sexually active individuals as in most papers on the subject. Parametrization of sexual contact patterns of sexually active individuals was undertaken by following the standard methodology for models for sexually transmitted infections, based on the "preferred mixing" approach for populations with heterogeneous sexual activity (S1, S3). In the standard 1-dimensional preferred mixing approach individuals of one sex (e.g. males) in a given risk group *i* (e.g. by age or level or sexual activity) mix with individuals of the other sex (females) in risk group *j* by reserving a fraction ε (the "preference" parameter) of their sexual partnerships to individuals in the same risk group while remaining partnerships are allocated at random among females from all risk groups, i.e. according to the proportionate mixing rule. When risk groups are characterised by several dimensions, e.g. in our model age and sexual activity level, multi-dimensional preferred mixing needs to be considered, where the preferred mixing matrices from each single dimension considered are compounded independently, each one with its specific preference parameter ε. Let the preference parameters for the two dimensions considered be denoted as ε_a (age) and ε_l (SAL) respectively. To parametrize sexual contacts, we carried out the following steps. First, female age- and SAL-specific marginal mean numbers of sexual partners per year were computed from the numbers of different partners had during the last year reported in survey data (Figure S3, left panel). Second, male marginal mean contacts rates are computed residually (Figure S3, right panel) using the female dominance criterion, based on the knowledge of female mean contact rates, the frequency of males and females in each risk group and the so called "sexual-mixing restraints" (S1). Individuals who had reported 0 or 1 sexual partners in the previous year were assigned to the low SAL group (84.7% of all females and 63.9% of all males); those who had reported 2 to 5 sexual partners in the previous year were assigned to the intermediate SAL group (14.7% of all females and 26.4 of all males); and those reporting 6 or more sexual partners in the previous year were assigned to the high SAL group (0.6% of all females and 9.7% of all males).

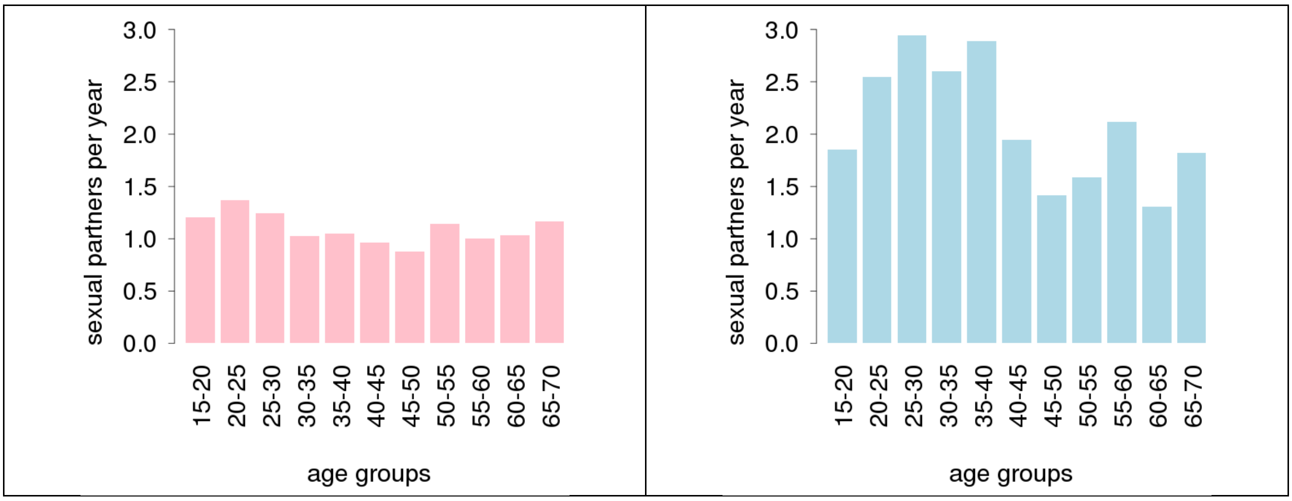

Figure S3. Observed (marginal) mean number of different sexual partners per year by 5-years age group. Left panel: females; right panel: males.

Finally, sex-specific preferred mixing matrices were parametrised using the "preferred mixing" approach (S1, S3). Mixing matrices are the Markov matrices relating age-SAL-specific marginal mean numbers of sexual partnerships with the corresponding joint mean number, as follows:

$$m_M[a_M, l_M, a_F, l_F] = c_M[a_M, l_M]\rho[a_M, a_F, l_M, l_F]$$

In substantive terms mixing matrices assign the proportions of partnerships that individuals having age-SAL (a,l) have with partners having age-SAL (a',l'). Using the "preferred mixing" approach the mixing matrices are defined as follows:

$$\rho[a_M, a_F, l_M, l_F] = \left(\varepsilon_a n_M[a_M] + (1-\varepsilon_a)\kappa_{a_F,a_M}\right)\left(\varepsilon_l u_M[a_M, l_M] + (1-\varepsilon_l)\kappa_{l_F,l_M}\right)$$

Where $n_M(a_M)$ is the fraction of men of age a_M over the total male population, $u_M(a_M, l_M)$ is the fraction of men of SAL l_M over the population of men of age a_M and $\kappa_{i,j}$ is Kronecker's discrete function, equal to 1 only for i =j and 0 elsewhere. The values of ε_a and ε_l were left as free model parameters and estimated by fitting to HPV prevalence data (see section S3 of this text).

S3. **Parametrization**

The region of exploration of the parameter space was defined by identifying plausible minimum and maximum values of each of the model's free parameters, through an extensive literature search (about 180 different studies were reviewed, of which 40 contained relevant data). These ranges are reported in Table S3 (only studies suggesting minimal or maximal values are cited).

Table S3: ranges of variability for free model parameters

Parameter	Unit of measure	Min	Ref	Max	Ref
Natural history parameters					
π_{FM}, π_{MF}	%	0	-	100	-
ε_a	%	0	-	100	-
ε_l	%	0	-	100	-
z	%	0	-	100	-
d = 1/γ	years	0.5	(S21)	2.4	(S22)
Progression/regression parameters					
H_{Y1}	1/year	0.02	(S23)	0.099	(S24)
H_{1X}	1/year	0.099	(S22)	2.564	(S25)
H_{12}	1/year	0.02	(S23)	0.675	(S25)
H_{2X}	1/year	0.018	(S23)	1.095	(S26)
H_{21}	1/year	0.117	(S26)	0.278	(S21)

H_{23}	1/year	0.02	(S27)	0.545	(S28)
H_{3X}	1/year	0.003	(S21)	0.095	(S23)
H_{32}	1/year	0.03	(S24)	0.055	(S29)
H_{3CIS}	1/year	0.013	(S23)	0.081	(S30)
H_{CIS-CC}	1/year	0.002	(S31)	0.05	(S32)

Poisson likelihood of the age-specific HPV prevalence

The estimation of natural history parameters was conducted by maximization of the Poisson likelihood of the age specific HPV prevalence during the first step of the parametrization procedure (see main text of this manuscript). The Poisson likelihood was calculated as:

$$L = \prod_i \frac{e^{-P_i} P_i^{D_i}}{D_i!}$$

Where P_i are model predictions for the HPV prevalence in age group i, and D_i is the corresponding observation for that age class.

Distribution of best-fitting parameters

Following (S33), we report in Figure S4 the distributions of best-fitting values of free model parameters, normalized with respect to the range of variability assumed from the literature search and summarized by boxplots representing the 95% CI, and the first, second and third quartiles.

While parameters related to the natural history of infection (the six leftmost boxplots in Figure S4) have a relatively narrow range, parameters accounting for progression and regression to different degrees of lesions and cancer have a much wider variability, probably due to the strong correlation between pairs of parameters and the lack of data to fit intermediate targets such as the prevalence of HPV16/18 lesions by age.

S4. Comparison between predictions from different best-fitting parameters

Given the relatively large variability in the estimates of progression/regression parameters, we show in Figure S5 the simulated predictions of cervical cancer incident cases produced by the 50 best-fitting parameter sets. In this analysis, we considered the two realistic vaccination schedules reported in the main text, i.e. the baseline vaccination of girls at 12 years and the baseline vaccination with additional catch-up at 25 years. In both scenarios predictions are qualitatively robust, with quantitative variations becoming less and less important with time. Predictions from the optimal parameter set (dashed line in Figure S5), presented in the main text, are overall conservative (i.e. suggest a higher number of cancer cases) with respect to the average of predictions obtained through different parameter configurations (solid line in Figure S5) in both vaccination scenarios.

Figure S4. Best-fitting parameter values, normalized to original range of variation.

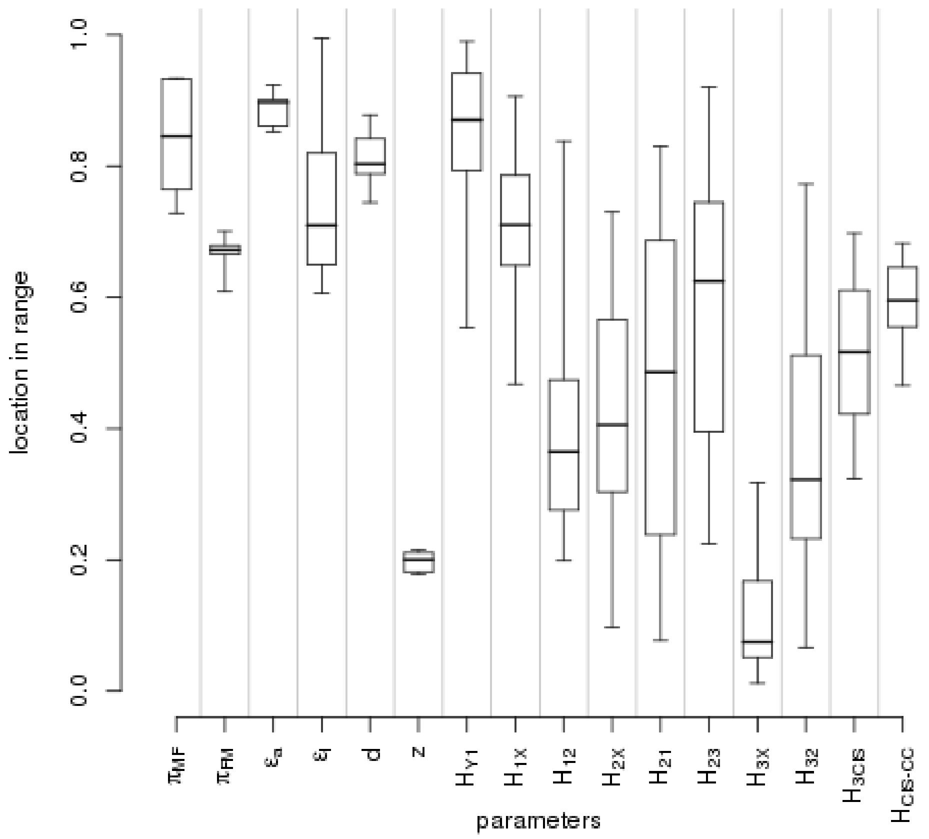

Figure S5. Variability of cervical cancer cases predicted over time by the 50 best-fitting parameter sets. Top: baseline scenario; bottom: catch-up scenario.

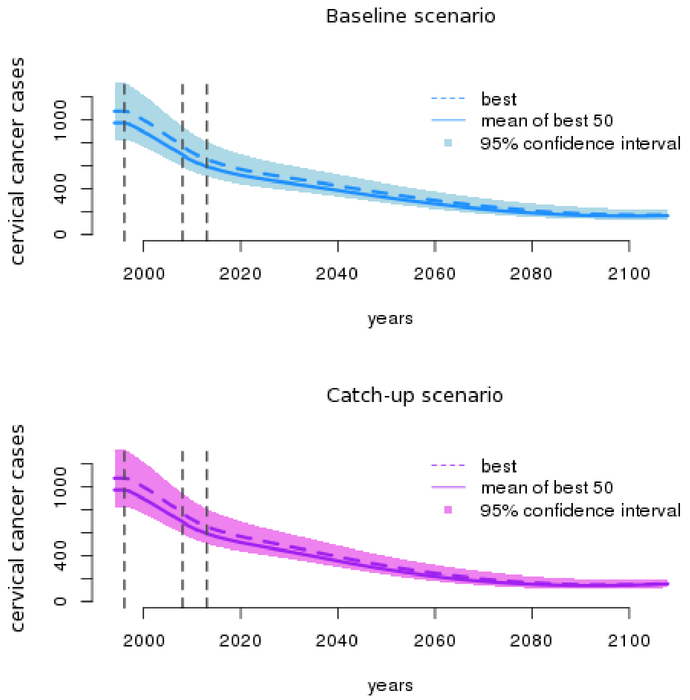

References

S1. Garnett GP, Anderson RM. (1993) Factors controlling the spread of HIV in heterosexual communities in developing countries: patterns of mixing between different age and sexual activity classes. Philos Trans R Soc Lond B Biol Sci. 342(1300):137-59.
S2. ISTAT (Italian Institute of Statistics). Available: http://dati.istat.it/?lang=en . Accessed: 10 September 2013.
S3. Elbasha EH, Dasbach EJ, Insinga RP. (2008) A multi-type HPV transmission model. Bull Math Biol 70(8):2126-76.
S4. Giambi C. Stato di avanzamento della campagna vaccinale per l'HPV: dati di copertura vaccinale al 30/06/2012 – Rapporto semestrale. Technical report of the National Public Health Institute. 2012.
S5. Schiller JT, Castellsagué X, Garland SM. (2012) A review of clinical trials of human papillomavirus prophylactic vaccines. Vaccine 30 Suppl 5:F123-38.
S6. David MP, Van Herck K, Hardt K, Tibaldi F, Dubin G, Descamps D, Van Damme P. (2009) Long-term persistence of anti-HPV-16 and -18 antibodies induced by vaccination with the AS04-adjuvanted cervical cancer vaccine: modeling of sustained antibody responses. Gynecol Oncol 115(3 Suppl):S1-6.
S7. Materia E, Rossi L, Spadea T, Cacciani L, Baglio G, Cesaroni G, Arcà M, Perucci CA. (2002) Hysterectomy and socioeconomic position in Rome, Italy. J Epidemiol Community Health 56 (6):461-5.
S8. Nobbenhuis MAE, Helmerhorst TJM, van den Brule AJC, Rozendaal L, Voorhorst FJ, Bezemer PD, Verheijen RHM, Meijer CJLM. (2001) Cytological regression and clearance of high-risk human papillomavirus in women with an abnormal cervical smear. Lancet. 358:1782-83
S9. Cruickshank ME, Sharp L, Chambers G, Smart L, Murray G. (2002) Persistent infection with human papillomavirus following the succesful treatment of high grade cervical intraepithelial neoplasia. BJOG 109:579-81.
S10. Paraskevaidis E, Arbyn M, Sotiriadis A, Diakomanolis E, Martin-Hirsch P, Koliopoulos G, Makrydimas G, Tofoski J, Roukos DH. (2004) The role of HPV DNA testing in the follow-up period after treatment for CIN: a systematic review of the literature. Cancer treatment reviews 30:205-11
S11. ONS (National Observatory on Screening). Yearly reports. Available: http://www.osservatorionazionalescreening.it/content/i-rapporti-annuali. Accessed: 10 September 2013.
S12. AIRTUM (Italian Association of Cancer Registries). Itacan Project. Available: http://itacan.ispo.toscana.it/English/itacan.htm. Accessed: 10 September 2013.
S13. Herbert A, Anshu A, Gregory M, Gupta SS, Singh N. (2009) Screen-detected invasive cervical carcinoma and its clinical significance during the introduction of organized screening. BJOG 116:854-59
S14. Martin-Hirsch P, Rash B, Martin A, Standaert B. (2007) Management of women with abnormal cervical cytology: treatment patterns and associated costs in England and Wales. BJOG 114(4):408-15.
S15. Szarewski A, Cuzick J, Edwards R, Butler B, Singer A. (1991) The use of cervicography in a primary screening service. BJOG 98(3):313-317.
S16. Cuzick J, Szarewski A, Cubie H, Hulman G, Kitchener H, Luesley D, McGoogan E, Menon U, Terry G, Edwards R, Brooks C, Desai M, Gie C, Ho L, Jacobs I, Pickles C, Sasieni P. (2003) Management of women who test positive for high-risk types of human papillomavirus: the HART study. Lancet. 362(9399):1871-6.
S17. Petry KU, Menton S, Menton M, van Loenen-Frosch F, de Carvalho Gomes H, Holz B, Schopp B, Garbrecht-Buettner S, Davies P, Boehmer G, van den Akker E, Iftner T. (2003) Inclusion of HPV testing in routine cervical cancer screening for women above 29 years in Germany: results for 8466 patients. British Journal of Cancer 88:1570-7.
S18. Dalla Palma P, Giorgi Rossi P, Collllina G, Buccoliero AM, Ghiringhello B, Lestani M, Onnis G, Aldovini D, Galanti G, Casadei G, Aldi M, Gomes V, Giubilato P, Ronco G, and the NTCC Pathology Group. (2008) The risk of false-positive histology according to the reason for colposcopy referral in cervical cancer screening. Am J Clin Pathol. 129-75-80.
S19. Stoler MH, Vichnin MD, Ferenczy A, Ferris DG, Perez G, Paavonen J, Joura EA, Djursing H, Sigurdsson K, Jefferson L, Alvarez F, Sings HL, Lu S, James MK, Saah A, Haupt RM. (2011) The accuracy of colposcopic biopsy: analyses from the placebo arm of the Gardasil clinical trials. International Journal of Cancer 128:1354-62.
S20. Hernes G. (1972) The process of entry into first marriage. American Sociological Review 37:173-82.
S21. Syrjanen K, Kataja V, Yliskoski M, Chang F, Syrjanen S, Saarikoski S. (1992) Natural history of cervical human papillomavirus lesions does not substantiate the biologic relevance of the Bethesda system. Obstetrics&Gynecology 79(5).
S22. Canfell K, Barnabas R, Patnick J, Beral V. (2004) The predicted effect of changes in cervical screening practice in the UK: results from a modelling study. British Journal of Cancer 91:530-6.

S23. Kataja V, Syrjanen K, Mantyarvi R, Vayrynen M, Syrjanen S, Saarikoski S, Parkkinen S, Yliskoski M, Salonen JT, Castren O. (1989) Prospective follow-up of cervical HPV infections: life table analysis of histopathological, cytological and colposcopic data. European Journal of Epidemiology 5(1):1-7.
S24. Insinga RP, Dasbach EJ, Elbasha EH, Liaw KL, Barr E. (2007) Incidence and duration of cervical human papillomavirus 6, 11, 16, and 18 infections in young women: an evaluation from multiple analytic perspectives. Cancer Epidemiol Biomarkers Prev 16(4):709-15.
S25. Shi JF, Belinson JL, Zhao FH, Pretorius RG, Li J, Ma JF, Chen F, Xiang W, Pan QJ, Zhang X, Zhang WH, Qiao YL, Smith JS. (2009) Human papillomavirus testing for cervical cancer screening: results from a 6-year prospective study in rural China. American Journal of Epidemiology 170(6):708-16.
S26. De Aloysio D, Miliffi L, Iannicelli T, Penacchioni P, Bottiglioni F. (1994) Intramuscular interferon-beta treatment of cervical intraepithelial neoplasia II associated with human papillomavirus infection. Acta Obstet Gynecol Scand 73(5):420-4.
S27. Moscicki AB, Ma Y, Wibbelsman C, Darragh TM, Powers A, Farhat S, Shiboski S. (2010) Rate and Risks for Regression of CIN-2 in adolescents and young women. Obstet Gynecol 116(6):1373-80.
S28. Discacciati MG, de Souza CAS, d'Otavianno MG, Angelo-Andrade LAL, Westin MCA, Rabelo-Santos SH, Zeferino LC. (2011) Outcome of expectant management of cervical intraepithelial neoplasia grade 2 in women followed for 12 months. European Journal of Obstetrics&Ginecology and Reproductive Biology 155:204-8.
S29. Furber SE, Weisberg E, Simpson JM. (1997) Progression and regression of low-grade epithelial abnormalities of the cervix. Aust. NZ J Obstet Gynaecol 37:107-112.
S30. Westergaard L, Norgaard M. (1981) Severe cervical dysplasia. Control by biopsies or primary conization? A comparative study. Acta Obstet Gynecol Scand 60(6):549-54.
S31. Goldie SJ, Kohli M, Grima D, Weinstein MC, Wright TC, Bosch FX, Franco E. (2004) Projected clinical benefits and cost-effectiveness of a human papillomavirus 16/18 vaccine. JNCI 96(8):604-15.
S32. Sanders GD, Taira AV. (2003) Cost Effectiveness of a Potential Vaccine for Human papillomavirus. Emerg Infect Dis 9(1):37-48.
S33. Van de Velde N, Boily MC, Drolet M, Franco EL, Mayrand MH, Kliewer EV, Coutlée F, Laprise JF, Malagón T, Brisson M. (2012) Population-level impact of the bivalent, quadrivalent, and nonavalent human papillomavirus vaccines: a model-based analysis. J Natl Cancer Inst 104(22):1712-23.
